# Dual Energy X-Ray Absorptiometry Compared with Anthropometry in Relation to Cardio-Metabolic Risk Factors in a Young Adult Population: Is the ‘Gold Standard’ Tarnished?

**DOI:** 10.1371/journal.pone.0162164

**Published:** 2016-09-13

**Authors:** Denise L. Demmer, Lawrence J. Beilin, Beth Hands, Sally Burrows, Craig E. Pennell, Stephen J. Lye, Jennifer A. Mountain, Trevor A. Mori

**Affiliations:** 1 School of Medicine and Pharmacology, Royal Perth Hospital Unit, The University of Western Australia, Perth, Australia; 2 Institute for Health Research, The University of Notre Dame Australia, Perth, Australia; 3 School of Women’s and Infants Health, The University of Western Australia, Perth, Australia; 4 Lunenfeld-Tanenbaum Research Institute, University of Toronto, Toronto, Canada; 5 School of Population Health, The University of Western Australia, Perth, Australia; Temple University School of Medicine, UNITED STATES

## Abstract

**Background and Aims:**

Assessment of adiposity using dual energy x-ray absorptiometry (DXA) has been considered more advantageous in comparison to anthropometry for predicting cardio-metabolic risk in the older population, by virtue of its ability to distinguish total and regional fat. Nonetheless, there is increasing uncertainty regarding the relative superiority of DXA and little comparative data exist in young adults. This study aimed to identify which measure of adiposity determined by either DXA or anthropometry is optimal within a range of cardio-metabolic risk factors in young adults.

**Methods and Results:**

1138 adults aged 20 years were assessed by DXA and standard anthropometry from the Western Australian Pregnancy Cohort (Raine) Study. Cross-sectional linear regression analyses were performed. Waist to height ratio was superior to any DXA measure with HDL-C. BMI was the superior model in relation to blood pressure than any DXA measure. Midriff fat mass (DXA) and waist circumference were comparable in relation to glucose. For all the other cardio-metabolic variables, anthropometric and DXA measures were comparable. DXA midriff fat mass compared with BMI or waist hip ratio was the superior measure for triglycerides, insulin and HOMA-IR.

**Conclusion:**

Although midriff fat mass (measured by DXA) was the superior measure with insulin sensitivity and triglycerides, the anthropometric measures were better or equal with various DXA measures for majority of the cardio-metabolic risk factors. Our findings suggest, clinical anthropometry is generally as useful as DXA in the evaluation of the individual cardio-metabolic risk factors in young adults.

## Introduction

The prevalence of obesity is increasing worldwide. In 2014 more than 1.9 billion adults, 18 years and older, were overweight and approximately 600 million adults were obese [1]. Excess body fat is an established risk factor for numerous chronic diseases and premature death [2, 3]. Most studies seeking to increase the understanding of the negative influence of obesity have been based on body mass index (BMI). However, BMI does not reflect total body adiposity because it cannot differentiate between lean and fat mass of an individual. Alternative measures such as waist circumference or waist-height ratio may be better clinical indicators of adiposity [4].

Dual energy x-ray absorptiometry (DXA) is the gold standard for the diagnosis of osteoporosis [5] and has increasingly been used for the diagnosis/management of overweight and obesity and in clinical research in cardiovascular disease [6–8]. The question arises as to whether the use of DXA in the management of overweight and obesity is justified in terms of cost and/or complexity, in return for any clear-cut scientific or clinical value. There are commercial drivers for the use of DXA; for example in the USA a patient can expect to pay approximately $100 US in the public health setting or $250 US within the private health sector for a DXA scan without rebate [9]. There has been a 65% decline in Medicare reimbursement of DXA bone mineral density testing in the non-facility setting, from approximately $140 in 2006 to $50 in 2014 [10]. At this lower level, providers of DXA scans are finding it more difficult to cover the operating costs, due to funding cuts [11]. Therefore, there has been some motivation to find another use for DXA that may be clinically relevant to health professionals, particularly to allow for early identification and intervention of individuals in relation to obesity and cardio-metabolic health.

The dominance and differential effects of DXA over anthropometry for estimating the presence of cardio-metabolic risk has not been clearly established. Studies in middle aged adults, which compare anthropometric and DXA adiposity measurements, have been inconsistent with respect to the strength of associations with cardio-metabolic risk factors, most likely due to methodological inconsistencies in defining adiposity [2, 12]. Moreover, many of these reports only take into account estimates of total body fat percentage and not fat distribution or abdominal fat mass ^10–14^. In addition, there is also a lack of reported data comparing DXA against cardio-metabolic risk factors solely in young adult populations [4, 12].

Obesity in young adults is an important predictor for subsequent coronary disease and diabetes in middle to old age [13, 14]. Australia follows worldwide trends showing increasing levels of obesity and Type 2 diabetes in early adulthood [15]. The early identification of individuals who are of increased risk of coronary disease and diabetes in later life has the potential to implement lifestyle modifications that could reduce this risk. This study therefore aimed to identify which measure of adiposity determined by either DXA (total body fat percentage, Fat Distribution Index and midriff fat mass) or anthropometry (abdominal skinfold, waist circumference, waist to height ratio, weight and BMI), is optimal within a range of cardio-metabolic risk factors in a sample of healthy young adults from the Western Australian Pregnancy Cohort (Raine) Study.

## Methods

### Participants

The Western Australian Pregnancy Cohort (Raine) Study is a prospective population study where pregnant women between 16 to 20 weeks gestation were recruited from King Edward Memorial Hospital and closely located practices. The mothers gave birth to 2868 live infants. Detailed information on the methods of the Raine Study has previously been reported [16]. The present population comprised 1273 adults from the Raine Study who attended the 20 year old survey. Written informed consent was obtained from the participants. Ethics approval for the 20 year assessment was obtained from the Human Research Ethics Committee at the University of Western Australia.

### Adult anthropometry and DXA measurements

At the 20 year follow up, height was measured by a wall mounted Stadiometer (to the nearest 0.1 cm) and weight was measured (to the nearest 100g) with participants dressed in light clothes. Body mass index (BMI) was calculated as weight (kg) divided by the square of height (m^2^). Waist circumference was evaluated at the umbilicus level and hip circumference at the level of the maximum posterior extension of the buttocks with a tape measure (to the nearest 0.1 cm). The abdominal skinfold was measured from a vertical skinfold immediately to the left of the umbilicus, the suprailiac skinfold was assessed from a diagonal fold located 1 cm above the anterior superior iliac crest and the tricep skinfold was measured from a vertical skinfold along the midline on the back of the triceps of the right arm using a skinfold calliper (Holtain, Crosswell, United Kingdom) [17]. All skinfold measurements were assessed twice with the average of the skinfolds calculated. The sum of three skinfolds was calculated from the abdominal, suprailiac and tricep skinfold sites and this characterises the subcutaneous fat thickness at different regions of the body [18]. Waist to hip and waist to height ratios were derived from the division of waist circumference (cm) by the hip circumference (cm) and height (m), respectively.

The study used a Norland XR-36 densitometer (Norland Medical Systems, Inc., Fort Atkinson, WI, USA) to provide estimates of whole body fat mass (g), lean mass (g), midriff fat mass (g) (vertebrae L1—L4). Total body fat percentage was estimated as total body fat mass (g) / total mass x 100. The Fat Distribution Index was calculated from the formula chest fat mass (g) + midriff fat mass (g) / pelvis fat mass (g) + left leg fat mass (g) + right leg fat mass (g) [19]. All measurements were performed by trained research personnel.

### Biochemistry and blood pressure measurements

Venous blood samples taken after an overnight fast were analysed in the PathWest Laboratory at Royal Perth Hospital for serum glucose, insulin, total cholesterol, triglycerides, high density lipoprotein-cholesterol (HDL-C) and high sensitivity C-reactive protein (hs-CRP) [20]. Low density lipoprotein-cholesterol (LDL-C) was calculated using the Friedewald equation [21]. The homeostatic model assessment of insulin resistance (HOMA-IR) was calculated using the formula: fasting insulin (μU/ml) x fasting glucose (mmol/L) / 22.5 [22]. BP was measured using an oscillometric sphygmomanometer with the appropriate cuff size for arm circumference (DINAMAP vital signs monitor 8100, DINAMAP XL vital signs monitor or DINAMAP ProCare 100; GE Healthcare). Six BP readings were obtained every 2 minutes within a 10 minute time period in the supine position, after a 5 minute resting period. The average BP value was calculated using the last five readings to obtain systolic and diastolic BP values [23].

### Statistical analysis

Characteristics of the sample were summarised using means and standard deviations (SD) separately for males and females. Differences between the sexes were tested using t-tests. All reported p values are 2-tailed and significance was set at α = 0.05.

Linear regression analyses examined the relationship between each adiposity measures and cardio-metabolic risk factors. All models included and tested the interaction between sex and the adiposity indicator. For each outcome, comparisons were made within the set of models, using each adiposity measure to identify the best DXA measure related to a given cardio-metabolic risk factor and similarly for the anthropometric measures. The measures identified as best from each of these two sets (DXA and anthropometry) were then compared. For example the model of DXA midriff fat mass and triglycerides was compared with the model of waist/height and triglycerides. Given that these models were not nested within each other, likelihood ratio tests were not appropriate to formally compare models. Thus the Akaike Information Criterion (AIC) was utilised [24]. As required, all models for a particular outcome were constructed on a static sample determined by complete data on all adiposity measures. For any given outcome, the model with the minimum AIC was deemed to be the best model. Differences from the minimum AIC provide a strength of evidence comparison and the ranking of models with respect to the best model. By calculating differences between AIC, arbitrary scaling constants are removed allowing the application of guidelines to interpret the difference. Models where the difference in AIC is ≤2 indicates substantial support that they are not different, while support for this claim decreases as the difference in AIC increases (AIC values above 10 are considered to have no support for equivalence). The adjusted R^2^ for each model was also reported. In addition to models with a single adiposity measure, combinations of anthropometric and DXA measures were explored in a similar manner. The combinations included midriff fat mass and waist circumference, fat distribution index and BMI, and midriff fat mass and BMI. Tobit regression (for censored data) was utilised for fasting insulin and hs-CRP due to the lower boundary of the test [25]. All other regressions performed were ordinary linear regression. Variables were log transformed if the values were not normally distributed (triglycerides, HDL-C, insulin, hs-CRP). Data were analysed using STATA (StataCorp, 2011. *Stata Statistical Software*: *release 12*. College Station. TX: StataCorp, LP).

## Results

### Descriptive characteristics

General characteristics of body composition using DXA, anthropometry and the individual cardio-metabolic risk factors are shown separately for females and males in Table 1.

**Table 1:** Descriptive characteristics of males and females from the Western Australian Pregnancy Cohort at 20 years of age.

Measure	Females	Males	p value
	n = 532	n = 606	
**DXA**			
Total body fat percentage (%)	39.3 (8.9)	21.8 (8.7)	<0.001
Fat distribution index (g)	17 318.2 (7 349.1)	11235.7 (6 431.0)	<0.001
Midriff fat mass (g)	1 401.7 (932.9)	1 091.0 (887.6)	<0.001
**Anthropometry**			
Abdominal skinfold (mm)	25.4 (8.6)	21.5 (10.2)	<0.001
Waist circumference (cm)	77.2 (13.0)	83.0 (12.2)	<0.001
Waist/height ratio	46.7 (8.0)	46.4 (6.6)	0.44
Height (m)	1.66 (0.1)	1.78 (0.1)	<0.001
Weight (kg)	67.1 (15.8)	78.7 (16.4)	<0.001
BMI (kg/m^2^)	24.4 (5.6)	24.5 (4.5)	0.71
**Biochemistry**			
Cholesterol (mmol/L)	4.5 (0.8)	4.2 (0.8)	<0.001
Triglycerides (mmol/L)	1.0 (0.5)	1.1 (0.6)	0.03
HDL-C (mmol/L)	1.4 (0.3)	1.2 (0.2)	<0.001
LDL-C (mmol/L)	2.6 (0.6)	2.4 (0.7)	<0.001
hs C-reactive protein (mg/L)	3.0 (5.5)	2.1 (5.8)	0.004
Glucose (mmol/L)	4.8 (0.4)	5.1 (0.4)	<0.001
Insulin (mU/L)	1.3 (0.7)	1.2 (0.7)	0.002
HOMA-IR	1.1 (1.2)	1.0 (1.4)	0.69
**Blood Pressure**			
Systolic BP (mmHg)	111.1 (10.2)	122.3 (11.8)	<0.001
Diastolic BP (mmHg)	65.4 (7.2)	65.2 (7.8)	0.62

Descriptive characteristics are presented as means and SD. BMI, body mass index; HDL, high-density lipoprotein; LDL, low-density lipoprotein; hs C-reactive protein; high sensitivity C-reactive protein; HOMA-IR, homeostatic model assessment of insulin resistance; BP, blood pressure.

Ethnicity in the cohort was predominantly Caucasian (93%). At 20 years of age, females had a higher total body fat percentage, fat distribution index and midriff fat mass compared with males (p <0.001) (Table 1). Males were taller, heavier and had a greater waist circumference than females (all p <0.001) but had a significantly lower abdominal skinfold. Males and females were not statistically different for waist to height ratio and BMI. All cardio-metabolic risk factors were statistically different between males and females except for HOMA-IR and diastolic BP. Females had lower systolic BP, triglycerides and glucose, and higher total cholesterol, HDL-C, LDL-C, insulin and hs-CRP, than males. The number of individuals in the sample with metabolic syndrome according to the revised National Cholesterol Education Program Adult Treatment Panel III guidelines [26] was 26 females (1.8%) and 47 males (3.3%).

### DXA and anthropometry measures with the individual cardio-metabolic risk factors

#### (i) Comparisons within DXA and anthropometric measures

Table 2 summarises the DXA and anthropometry measures that produced the lowest AIC for each of the individual cardio-metabolic risk factors. No statistically significant differences between the sexes in the relationship between the adiposity measures and the cardio-metabolic risk factors were found for any outcome. The interaction term was therefore removed and a single adiposity coefficient applicable to both males and females was estimated. Within the DXA measures, midriff fat mass was the superior model for all risk factors with the exception of HDL-C, hs-CRP and systolic BP. In contrast, there was considerable variation in the best anthropometric measure across the cardio-metabolic risk factors: BMI was the best measure for systolic BP, hs-CRP, insulin and HOMA-IR; waist to height ratio was best for triglycerides, HDL-C and LDL-C; and abdominal skin fold was best for cholesterol and diastolic BP. Body adiposity index and sum of skinfolds, as anthropometry measurements, were not evident to have the lowest AIC with any of the risk factors. None of the combinations of DXA with anthropometry measures was superior to the individual DXA and anthropometry measures with any of the cardio-metabolic risk factors.

**Table 2:** The Akaike Information Criterion differences between the best DXA and best anthropometry measures with the individual cardio-metabolic risk factors in young adults.

Adiposity indices	Best DXA	Best	AIC difference
		Anthropometry	(DXA –
	AIC R2	AIC R2	Anthropometry)
**Cholesterol**	Midriff fat mass	Abdominal skinfold	
N = 1021	2325.3 0.08	2327.73 0.04	-2.46
**Triglycerides**	Midriff fat mass*	Waist/height ratio	
N = 1021	1550.2 0.10	1560.64 0.09	-10.49
**HDL-C**	Fat distribution	Waist/height ratio*	
	index		
N = 1021	343.3 0.19	331.78 0.20	11.54
**LDL-C**	Midriff fat mass	Waist/height ratio	
N = 1021	1972.1 0.05	1971.55 0.06	0.52
**hs C-reactive protein**	Total body fat	BMI	
	percentage		
N = 1021	6359.9 0.006	6362.34 0.005	-2.41
**Glucose**	Midriff fat mass	Waist circumference	
N = 1021	957.8 0.09	958.08 0.09	-0.28
**Insulin**	Midriff fat mass*	BMI	
N = 1021	2158.3 0.08	2168.08 0.07	-9.74
**HOMA-IR**	Midriff fat mass*	BMI	
N = 1021	3261.1 0.04	3270.46 0.04	-9.36
**Systolic BP**	Fat distribution	BMI*	
	index		
N = 1180	8884.8 0.27	8831.09 0.30	53.7
**Diastolic BP**	Midriff fat mass	Abdominal skinfold	
N = 1180	8043.4 0.03	8043.73 0.003	-0.38

*Best adiposity measure

AIC and R^2^ values were derived from linear or Tobit regression models adjusted for sex (S1–S3 Tables). AIC differences were derived from the best DXA AIC value—the best anthropometry AIC value. Differences in AIC between models of approximately 2 were considered to indicate equivalent adiposity measures. Otherwise the model with the lowest AIC was considered superior.

AIC, Akaike information criterion; HDL-C, high-density lipoprotein cholesterol; LDL-C, low-density lipoprotein cholesterol; HOMA-IR, homeostatic model assessment of insulin resistance; hs C-reactive protein; high sensitivity C-reactive protein; BP, blood pressure; BMI, body mass index; WC, waist circumference.

#### (ii) Comparisons between the best DXA and best anthropometry measure

Differences between the best DXA and anthropometry measure for individual cardio-metabolic risk factors either clearly indicated the dominance of one measure (delta AIC >10) or suggested equivalence (delta AIC ≤2) (Table 2). The model that incorporated midriff fat mass was superior for triglycerides, insulin and HOMA-IR. In contrast, waist-to-height ratio was the superior model in relation to HDL-C and BMI was superior to any DXA measure in relation to systolic BP. For all the other cardio-metabolic variables, anthropometric and DXA measures were comparable. The adjusted R-squared values show marginal differences between the best DXA and anthropometry measures.

## Discussion

In this population cohort of young adults, clinical anthropometry measures either outperformed or were equivalent to DXA for majority of the cardio-metabolic risk factors. The exception was DXA midriff fat mass as it was superior for triglycerides, insulin and HOMA-IR. Combinations of various DXA and anthropometry measures were no better than the individual DXA or anthropometry. While not reported here, we found no additional value of lean body mass measurements or a fat/lean mass ratio compared with clinical anthropometry. Our results show the utility of anthropometry as a simple, cost-effective primary screening tool for the identification of individuals at risk. Our data, however, do not preclude the use of DXA in relation to more focussed research questions or for a further detailed clinical assessment of patients.

It is difficult to justify high equipment and scanning costs, burden on the individual and reduced accessibility imposed by DXA used as a primary adiposity screening tool in a clinical setting or for population level research into cardio-metabolic disease. In addition, several factors affecting the efficacy of DXA, include the interpretation of DXA scans which require specialised training, technician error operating equipment, the type of clothing and positioning of the patient on the table. With worldwide health costs rapidly increasing, the use of DXA for fat assessment is hard to justify, except in relation to a much more focussed research question and for a more detailed clinical assessment of the patient, as a secondary screening tool. In this latter setting, other direct imaging methods such as magnetic resonance imaging, computer tomography, ultrasound and possibly bioelectrical impedance analysis techniques can be useful for the quantification and differentiation of subcutaneous and visceral fat tissue [27–29].

In our study, we showed that the much criticised BMI was the best measure with systolic BP and was superior to any DXA derived measure. Height and weight were also important individual determinants of BP; however, neither was better than BMI in relation to systolic BP. In other studies, BMI was significantly related to systolic BP among US adolescents [4] [30, 31], the NHANES study in children found BMI was more strongly correlated with systolic BP than DXA fat mass percentage [32]. Ito et al [33] reported the accuracy of detecting hypertension and dyslipidaemia was comparable between BMI, waist circumference and waist to height ratio measures and the DXA measures of total percentage fat mass and midriff fat mass percentage in adults.

We found no other comparisons of DXA and anthropometry in relation to wide range of cardio-metabolic risk factors in young adults from population based studies. Studies in middle aged adults and children have also shown lack of dominance of DXA over BMI. Krachler et al [30] found BMI had similar predictive power compared to DXA fat mass percentage and bio-impedance analysis for hypertension, impaired fasting glucose, dyslipidaemia and the metabolic syndrome, in middle aged adults. BMI and waist circumference were similarly correlated with DXA fat mass and fat mass percentage in relation to hs-CRP, BP and fasting lipids, glucose and insulin in adults [31] and in youth aged 8–18 years of age [32]. Percentage body fat (DXA) did not produce stronger associations in estimating components of the metabolic syndrome [34] and cardiovascular risk factors than BMI and skinfold thickness in youth [35]. Our findings on hs-CRP contrast with those of Vega et al [36] who observed that DXA total body fat percentage was better correlated with hs-CRP than BMI, in middle-aged US adults.

The strengths of this study include data from a large sample population within a narrow age range and a breadth of measures evaluating adiposity using DXA and anthropometry. In addition, we examined an array of cardio-metabolic risk factors, in contrast to most other studies which had a narrower focus. A robust statistical approach was used to compare the performance of the adiposity measures within each outcome. Limitations of this study include its cross-sectional nature and the DXA instrument utilised cannot distinguish between visceral adipose tissue and subcutaneous fat adipose tissue. Therefore, we could not separately analyse these two components of adiposity. Additionally, the Norland software used in this study does not differentiate between abdominal visceral and subcutaneous fat. However, as the Hologic DXA software can differentiate between these compartments and our findings may not be applicable to all DXA machine models.

Overall our findings add to the weight of evidence suggesting that anthropometry measures are generally as useful as DXA in the evaluation of the individual cardio-metabolic risk factors. Anthropometry offers the advantages of technical simplicity, convenience, and a lower cost compared with DXA. Anthropometry has a great utility as a cost effective primary screening tool of excess adiposity and allows for the early identification of individuals at risk.

## Supporting Information

S1 Table Adjusted R squared and Akaike Information Criterion between DXA and anthropometry adiposity measures with lipid, lipoprotein and inflammatory cardio-metabolic risk factors in young adults. Adjusted R^2^ and AIC values were derived from linear or Tobit regression models and was adjusted for sex. AIC, Akaike information criterion; HDL-C, high-density lipoprotein cholesterol; LDL-C, low-density lipoprotein cholesterol; hs C-reactive protein; high sensitivity C-reactive protein; BMI, body mass index; WC, waist circumference. ^#^ Best adiposity measure; * Equivalent adiposity measure.
(PDF)

S2 Table Adjusted R squared and Akaike Information Criterion between DXA and anthropometry adiposity measures with insulin resistance cardio-metabolic risk factors in young adults. Adjusted R^2^ and AIC values were derived from linear or Tobit regression models and was adjusted for sex. AIC, Akaike information criterion; HOMA-IR, homeostatic model assessment of insulin resistance; BMI, body mass index; WC, waist circumference. ^#^ Best adiposity measure; * Equivalent adiposity measure.
(PDF)

S3 Table Adjusted R squared and Akaike Information Criterion between DXA and anthropometry adiposity measures with blood pressure cardio-metabolic risk factors in young adults. Adjusted R^2^ and AIC values were derived from linear regression models and was adjusted for sex. AIC, Akaike information criterion; SBP, systolic blood pressure; DBP, diastolic blood pressure; BMI, body mass index; WC, waist circumference. ^#^Best adiposity measure^; *^ Equivalent adiposity measure.
(PDF)
