# Correction: Dual Energy X-Ray Absorptiometry Compared with Anthropometry in Relation to Cardio-Metabolic Risk Factors in a Young Adult Population: Is the 'Gold Standard' Tarnished?

**DOI:** 10.1371/journal.pone.0168961

**Published:** 2016-12-20

**Authors:** Denise L. Demmer, Lawrence J. Beilin, Beth Hands, Sally Burrows, Kay L. Cox, Craig E. Pennell, Stephen J. Lye, Jennifer A. Mountain, Trevor A. Mori

Dr. Kay L. Cox should be included in the author byline. She should be listed as the fifth author, and her affiliation is #1: School of Medicine and Pharmacology, Royal Perth Hospital Unit, The University of Western Australia, Perth, Australia. The contributions of this author are as follows: Writing–review & editing.

Authors Beth Hands, Kay L. Cox and Jennifer A. Mountain should be noted as contributing equally to this work.

The correct citation is: Demmer DL, Beilin LJ, Hands B, Burrows S, Cox KL, Pennell CE, et al. (2016) Dual Energy X-Ray Absorptiometry Compared with Anthropometry in Relation to Cardio-Metabolic Risk Factors in a Young Adult Population: Is the ‘Gold Standard’ Tarnished? PLoS ONE 11(9): e0162164. doi:10.1371/journal.pone.0162164
