# Supplementary material for: Dual Energy X-Ray Absorptiometry Compared with Anthropometry in Relation to Cardio-Metabolic Risk Factors in a Young Adult Population: Is the ‘Gold Standard’ Tarnished?
Source: PLoS One. 2016 Sep 13;11(9):e0162164. doi: 10.1371/journal.pone.0162164 (PMC5021262; doi:10.1371/journal.pone.0162164)
Supplement: S1 Table — Adjusted R2 and AIC values were derived from linear or Tobit regression models and was adjusted for sex. AIC, Akaike information criterion; HDL-C, high-density lipoprotein cholesterol; LDL-C, low-density lipoprotein cholesterol; hs C-reactive protein; high sensitivity C-reactive protein; BMI, body mass index; WC, waist circumference. # Best adiposity measure; * Equivalent adiposity measure. (PDF) [file pone.0162164.s001.pdf]

S1 Table. Adjusted R squared and Akaike Information Criterion between DXA and anthropometry adiposity measures with lipid, lipoprotein and inflammatory cardiovascular risk factors in young adults.

Adiposity indices	Cholesterol N= 1021		Triglycerides N= 1021		HDL-C N= 1021		LDL-C N= 1021		C-reactive protein N= 1021	
	R^2	AIC	R^2	AIC	R^2	AIC	R^2	AIC	R^2	AIC
**DXA**										
Total body fat percentage	0.074	2330.3	0.073	1583.58	0.171	371.58	0.056	1972.69	**0.006**	**6359.93**
Fat distribution index	0.065	2340.43	0.079	1577.47	**0.193**	**343.32**	0.049	1980.38	0.006	6361.14
Midriff fat mass	**0.079**	**2325.27**	**0.103**	**1550.15**	0.19	347.88	**0.057**	**1972.07**	0.005	6363.48
**Anthropometry**										
Abdominal skinfold	**0.036**	**2327.73**	0.046	1590.85	0.348	376.37	0.032	1974.03	0.003	6374.39
Waist circumference	0.064	2341.83	0.087	1567.92	0.202	332.59	0.047	1982.84	0.004	6371.29
Waist/height ratio	0.073	2331.49	**0.094**	**1560.64**	**0.203**	**331.78**	**0.057**	**1971.55**	0.005	6365.29
Weight	0.051	2355.41	0.062	1596.28	0.188	350.54	0.032	1998.86	0.004	6373.67
BMI	0.066	2339.62	0.085	1570.97	0.201	334.26	0.05	1978.92	**0.005**	**6362.34**
**Combination**										
Midriff fat mass & WC	0.071	2399.91	0.090	1600.68	0.194	371.319	0.060	2033.51	0.005	6510.40
Fat distribution index & BMI	0.062	2409.68	0.071	1621.28	0.202	362.148	0.054	2040.47	0.006	6507.64
Midriff fat mass & BMI	0.069	2401.83	0.089	1601.07	0.201	363.06	0.059	2035.17	0.005	6509.48
**Best DXA model**	**Midriff fat mass***		**Midriff fat mass[#]**		**Fat distribution index**		**Midriff fat mass[*]**		**Total body fat percentage[#]**	

Best anthropometry model	Abdominal skinfold*	Waist height ratio	Waist height ratio#	Waist height ratio*	BMI

Adjusted R^2 and AIC values were derived from linear or Tobit regression models and was adjusted for sex. AIC, Akaike information criterion; HDL-C, high-density lipoprotein cholesterol; LDL-C, low-density lipoprotein cholesterol; hs C-Reactive Protein; high sensitivity C-Reactive Protein; BMI, body mass index; WC, waist circumference.

Best adiposity measure

* Equivalent adiposity measure
