# Supplementary material for: Dual Energy X-Ray Absorptiometry Compared with Anthropometry in Relation to Cardio-Metabolic Risk Factors in a Young Adult Population: Is the ‘Gold Standard’ Tarnished?
Source: PLoS One. 2016 Sep 13;11(9):e0162164. doi: 10.1371/journal.pone.0162164 (PMC5021262; doi:10.1371/journal.pone.0162164)
Supplement: S2 Table — Adjusted R2 and AIC values were derived from linear or Tobit regression models and was adjusted for sex. AIC, Akaike information criterion; HOMA-IR, homeostatic model assessment of insulin resistance; BMI, body mass index; WC, waist circumference. # Best adiposity measure; * Equivalent adiposity measure. (PDF) [file pone.0162164.s002.pdf]

**S2 Table. Adjusted R squared and Akaike Information Criterion between DXA and anthropometry adiposity measures and insulin resistance cardiovascular risk factors in young adults.**

| **Adiposity indices** | **Glucose N= 1021** | | **Insulin N= 1021** | | **HOMA-IR N= 1021** | |
|---|---|---|---|---|---|---|
| | $R^2$ | AIC | $R^2$ | AIC | $R^2$ | AIC |
| **DXA** | | | | | | |
| Total body fat percentage | 0.085 | 968.29 | 0.075 | 2173.49 | 0.027 | 3317.77 |
| Fat distribution index | 0.093 | 959.49 | 0.08 | 2160.63 | 0.036 | 3287.02 |
| Midriff fat mass | **0.094** | **957.8** | **0.081** | **2158.34** | **0.044** | **3261.1** |
| **Anthropometry** | | | | | | |
| Abdominal skinfold | 0.089 | 971.6 | 0.072 | 2179.52 | 0.023 | 3331.57 |
| Waist circumference | **0.094** | **958.08** | 0.07 | 2184.6 | 0.034 | 3295.29 |
| Waist/height ratio | 0.091 | 961.16 | 0.073 | 2177.8 | 0.037 | 3285.04 |
| Weight | 0.091 | 961.59 | 0.063 | 2201.3 | 0.031 | 3304.46 |
| BMI | 0.093 | 959.68 | **0.077** | **2168.08** | **0.041** | **3270.46** |
| **Combination** | | | | | | |
| Midriff fat mass & WC | 0.042 | 1766.41 | 0.081 | 2214.36 | 0.037 | 3357.34 |
| Fat distribution index & BMI | 0.045 | 1763.88 | 0.080 | 2216.47 | 0.034 | 3370.36 |
| Midriff fat mass & BMI | 0.044 | 1764.69 | 0.082 | 2211.78 | 0.038 | 3354.55 |
| **Best DXA model** | **Midriff fat mass*** | | **Midriff fat mass#** | | **Midriff fat mass#** | |
| **Best anthropometry model** | **Waist circumference*** | | **BMI** | | **BMI** | |

Adjusted $R^2$ and AIC values were derived from linear or Tobit regression models and was adjusted for sex. AIC, Akaike information criterion; HOMA-IR, homeostatic model assessment of insulin resistance; BMI, body mass index; WC, waist circumference.

# Best adiposity measure

* Equivalent adiposity measure
