# Supplementary material for: Dual Energy X-Ray Absorptiometry Compared with Anthropometry in Relation to Cardio-Metabolic Risk Factors in a Young Adult Population: Is the ‘Gold Standard’ Tarnished?
Source: PLoS One. 2016 Sep 13;11(9):e0162164. doi: 10.1371/journal.pone.0162164 (PMC5021262; doi:10.1371/journal.pone.0162164)
Supplement: S3 Table — Adjusted R2 and AIC values were derived from linear regression models and was adjusted for sex. AIC, Akaike information criterion; SBP, systolic blood pressure; DBP, diastolic blood pressure; BMI, body mass index; WC, waist circumference. #Best adiposity measure; * Equivalent adiposity measure. (PDF) [file pone.0162164.s003.pdf]

S3 Table. Adjusted R squared and Aikaike Information Criterion between DXA and anthropometry adiposity measures and blood pressure cardiovascular risk factors in young adults

**Adiposity indices**	**SBP N= 1180**		**DBP N= 1180**	
	R^2	AIC	R^2	AIC
**DXA**				
Total body fat percentage	0.248	8919.09	0.024	8048.48
Fat distribution index	**0.27**	**8884.79**	0.023	8050.01
Midriff fat mass	0.267	8889.3	**0.028**	**8043.35**
**Anthropometry**				
Abdominal skinfold	0.039	8880.85	**0.003**	**8043.73**
Waist circumference	0.295	8844.04	0.024	8048.48
Waist/height ratio	0.288	8855.11	0.023	8049.39
Weight	0.297	8839.28	0.012	8062.54
BMI	**0.302**	**8831.09**	0.014	8060.25
**Combination**				
Midriff fat mass & WC	0.2647	9102.196	0.0250	8227.364
Fat distribution index & BMI	0.2827	9072.438	0.0214	8231.764
Midriff fat mass & BMI	0.2832	9071.548	0.0262	8225.832
**Best DXA model**	**Fat distribution index**		**Midriff fat mass***	
**Best anthropometry model**	**BMI#**		**Abdominal skinfold***	

Adjusted R^2 and AIC values were derived from linear regression models and was adjusted for sex. AIC, Akaike information criterion; SBP, systolic blood pressure; DBP, diastolic blood pressure; BMI, body mass index; WC, waist circumference.

#Best adiposity measure

* Equivalent adiposity measure
